# Magnetic order and disorder environments in superantiferromagnetic $\hbox {NdCu}_{\mathbf{2}}$ nanoparticles

**DOI:** 10.1038/s41598-022-13817-7

**Published:** 2022-06-13

**Authors:** E. M. Jefremovas, P. Svedlindh, F. Damay, D. Alba Venero, A. Michels, J. A. Blanco, L. Fernández Barquín

**Affiliations:** 1 Department CITIMAC, Facultad de Ciencias, Universidad de Cantabria, 39005 Santander, Spain; 2 Department of Materials Science and Engineering, Uppsala University, Box 35, 751 03 Uppsala, Sweden; 3 Laboratoire Léon Brillouin, Université Paris–Saclay, CEA–CNRS, 91191 Gif–sur–Yvette Cedex, France; 4 ISIS Neutron and Muon Facility, Rutherford Appleton Laboratory, Didcot, OX11 0QX UK; 5 Department of Physics and Materials Science, University of Luxembourg, 1511 Luxembourg, Luxembourg; 6 Department of Physics, University of Oviedo, 33007 Oviedo, Spain

**Keywords:** Magnetic properties and materials, Nanoparticles, Structural properties

## Abstract

Magnetic nanoparticles exhibit two different local symmetry environments, one ascribed to the core and one corresponding to the nanoparticle surface. This implies the existence of a dual spin dynamics, leading to the presence of two different magnetic arrangements governed by different correlation lengths. In this work, two ensembles of $\hbox {NdCu}_{2}$ nanoparticles with mean sizes of 18 nm and 13 nm have been produced to unravel the magnetic couplings established among the magnetic moments located within the core and at the nanoparticle surface. To this end, we have combined neutron diffraction measurements, appropriate to investigate magnetically-ordered spin arrangements, with time-dependent macroscopic AC susceptibility measurements to reveal memory and aging effects. The observation of the latter phenomena are indicative of magnetically-frustrated states. The obtained results indicate that, while the $\hbox {Nd}^{3{+}}$ magnetic moments located within the nanoparticle core keep the bulk antiferromagnetic commensurate structure in the whole magnetic state, the correlations among the surface spins give rise to a collective frustrated spin-glass phase. The interpretation of the magnetic structure of the nanoparticles is complemented by specific-heat measurements, which further support the lack of incommensurability in the nanoparticle state.

## Introduction

Magnetically-frustrated systems constitute a vast and fascinating research topic in condensed-matter physics. Terms such as disorder, competing interactions, randomness, or broken inversion symmetry are at the heart of many complex magnetic phenomena and spin textures, as they are encountered e.g., in spin glasses (SG), spin ices, spin liquids, pyrochlore oxides, multiferroics, or skyrmion crystals^1–17^. In recent years, ensembles of magnetic nanoparticles (MNPs), where structural and ensuing magnetic disorder is inherently present due to the finite particle size, are attracting growing attention owing to their enormous potential for technological applications (see, e.g., Refs.^18–22^ and references therein). In particular, the spin dynamics and collective excitations of MNPs are the subject of intensive studies, where the antiferromagnetic (AF) systems stand out due to their relevance for data-storage applications and for the emerging fields of skyrmionics and spintronics^23–35^.

MNPs combine, in one single system, two different spin dynamics ascribed to two different symmetry environments—the particle core and the surface. Generally, it is found that the symmetry environment and the coordination number of the magnetic moments located within the nanoparticle core remain essentially the same as the ones found in the bulk counterpart^36^. For the surface spins, however, the situation is significantly different due to the existence of a non-negligible lattice distortion (strain) and reduced coordination numbers^37–39^. As a result, the magnetic interactions among surface spins will be changed and even frustrated, yielding disordered spin arrangements in the vicinity of the surface. By this token, minimal unit cell distortions might account for the switching between different types of magnetic orderings, such as, ferromagnetic (FM) and AF long-range orders akin to the achievement of an uncompensated magnetic moment or net magnetic signal found in $\hbox {Cr}_{2}\hbox {O}_{3}$^40^, NiO^41^, and CoO–Pt^42^. However, much less attention has been paid to the case of nanoparticles based on rare-earth elements.

In this work, we use neutron diffraction, AC susceptibility, and specific-heat measurements to investigate the structure and spin dynamics of two ensembles of $\hbox {NdCu}_{2}$ MNPs with average particle sizes of 18 nm and 13 nm. Previous investigations have showed that $\hbox {NdCu}_{2}$ hosts a complex magnetic phase diagram in the bulk state, with AF-coupled magnetic moments along the *a*-direction and FM-coupled *b*–*c* planes (with a Néel temperature of $T_N = 6.5$ K)^43^. In the low-temperature AF phase, a commensurate square-up modulation is established, whereas the rise in temperature provokes slight deviations from the equilibrium positions of the FM-coupled magnetic moments. At $T_R = 4.5$ K a reorientation of the magnetic moments takes place, and an incommensurate structure is present up to $T_{N}$. Concerning $\hbox {NdCu}_{2}$ MNPs, these have evidenced a superantiferromagnetic (SAF) structure, where (at $T = 1.5$ K) the *core* magnetic moments exhibit the commensurate square-up modulation of bulk $\hbox {NdCu}_{2}$^22,39,44^. By contrast, the magnetic moments located at the *surface* feature a collective freezing mechanism, following a SG dynamics. This AF-core/SG-surface arrangement is similar to the one reported for $\hbox {TbCu}_{2}$^22,44^, $\hbox {GdCu}_{2}$^38^, $\hbox {Cr}_{2}\hbox {O}_{3}$, NiO or CoO–Pt MNPs^40–42^. However, it is important to point out that, while for the case of the 3*d*-based MNPs, large exchange bias effect has been reported (up to several hundred mT^40–42,45,46^), the magnitude of the exchange bias in the 4*f*-based is remarkably reduced by two orders of magnitude (see Fig. S5 included in Supplementary Section “Exchange bias effect” in Ref.^47^). This evidences the enhanced role that the magnetically-disordered surface moments play into the magnetic dynamics of the system. These magnetically-disordered moments could lead to a modification of the complex AF structure that takes place within the nanoparticle core. Therefore, the microscopic analyses presented here will be focused on addressing the question whether the aforementioned commensurate-incommensurate transition also takes place in the MNP regime. Besides, we will evaluate quantitatively the surface disorder by evaluating the robustness of the collective SG state. For this purpose, the time-dependent magnetic susceptibility response will be analyzed. This approach, which has been employed previously to study the well-known power-law scaling of the critical behavior close to an anticipated SG temperature^48–55^, allows for the interpretation of subtle (very low magnetic signal) dynamic responses depending on the magnetic history (memory effects) and the time-dependent susceptibility (aging).

## Results and discussion

### Microscopic structural characterization: neutron diffraction and small-angle neutron scattering

**Figure 1:**
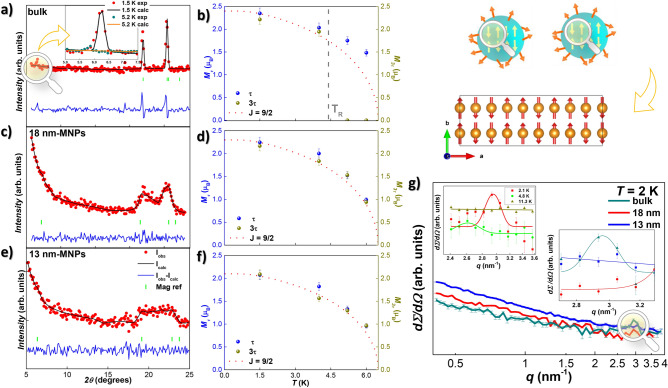
Neutron diffraction results. (**a**, **c**, **e**)  Experimental neutron diffraction patterns at $T = 5.2$ K (red dots), together with the Rietveld refinements (black lines) measured for bulk $\hbox {NdCu}_{2}$, 18 nm and 13 nm-sized MNPs, respectively. Inset in (**a**) zooms the commensurate (200) magnetic reflection, present at $T = 1.5$ K, and absent at $T=5.2$ K. (**b**, **d**, **f**): Temperature dependence of the magnetic moment *M* per unit cell. The position of the commensurate-to-incommensurate transition temperature is only marked for the bulk alloy [vertical gray dashed line in (**b**)]. (**g**) Differential SANS cross section $d\Sigma /d\Omega$ as a function of the momentum transfer *q* (log–log scale) measured for bulk (dark cyan), 18 nm (red), and 13 nm-sized (blue) $\hbox {NdCu}_{2}$ MNPs at zero field and at $T=2$ K (commensurate AF phase). Right inset zooms into the region nearby 2.6 $\lesssim q \lesssim$ 3.3 nm$^{-1}$ at $T = 2$ K. This region has been studied at $T = 2.1$ K and 4.8 K (incommensurate AF phase) and at 11.3 K (paramagnetic phase) for the bulk alloy (top left inset). Sketches of the MNPs and of commensurate magnetic structure are showed on the top right.

The nonmagnetic neutron diffraction (ND) (above $T_{N}$) and the X-ray diffraction patterns have been discussed previously (Ref.^39^). This study showed that the bulk orthorhombic *Imma* structure is maintained also at the MNPs, with lattice parameters close to those of the bulk alloy reported in Ref.^56^. For the MNPs, Rietveld refinements indicated a mean nanoparticle size of $\langle D \rangle =18.3(1.0)$ and 13.0(5) nm for 2h and 5h-milled MNPs; together with microstrain $\eta$ values of $\eta = 0.62(7) \%$ and $\eta = 0.59(1) \%$, respectively^39^. These values agree with the ones obtained from the nuclear magnetic structure, where $\langle D \rangle = 16.5(4)$ nm and 12.6(6) nm, respectively, as with the ones pointed from TEM characterization (see Supplementary Fig. S1 in Ref.^57^). Given the existence of this previous study, in this paper we will focus on the variation of the magnetic moment on the $\hbox {Nd}^{3{+}}$ sites. Figure 1a, c, and e include the ND patterns together with the Rietveld refinements (Bragg factor $R_{B} < 10 \, \%$ in all of the cases) for the three $\hbox {NdCu}_{2}$ alloys (bulk, 18 nm, and 13 nm) measured at $T = 5.2$ K (i.e., in the region between $T_{R}$ and $T_{N}$). Particularly, we want to focus on the region between $5^{\circ }< 2\theta < 25^{\circ }$, as this angular range encompasses only magnetic reflections. The complete diffractograms can be inspected in Supplementary Fig. S2 in Ref.^57^. The indicated values of the MNP sizes inserted in Fig. 1c and e, 18 nm and 13 nm, respectively, have been obtained by means of Rietveld refinements of the pattern in the paramagnetic region at $T = 10$ K^39^.

The magnetic unit cell of the bulk alloy included in Fig. 1a is well described using the propagation vector $\tau = (0.612, 0.042, 0)$ (i.e., an incommensurate description). No higher harmonics are detected within the experimental resolution. Although the Rietveld refinements indicate a description with a pure incommensurate phase to be the most suitable for the magnetic cell arrangement, a minor fraction of the magnetic moments (below 5%) still keeps the commensurate modulation, just in the same way as reported in Ref.^43^. The survival of the commensurate structure above $T_R$ can be clearly detected thanks to the emergence of a peak located at $2\theta \sim 6.3^{\circ }$ (blue-shadowed region), which corresponds to the (200) Bragg reflection of the commensurate arrangement. The huge reduction of the magnetic commensurate moments at $T = 5.2$ K accounts for the drop in the peak intensity compared to the situation at $T = 1.5$ K, as it can be observed in the inset. In the case of the MNPs, this reflection is masked, as there is an increasing scattering signal below $2\theta \sim 7^{\circ }$ ($q < 0.316\,\text{\AA} ^{-1}$). The rise of the magnetic intensity in the MNPs comes as a consequence of the incipient interparticle correlations, which has also been found in other SAF $\hbox {RCu}_{2}$ MNPs^22,39,44^.

On the other hand, the description of the magnetic unit cell corresponding to the MNPs keeps the commensurate arrangement up to $T_{N}$, as it can be observed according to the Rietveld refinements included in Fig. 1c and e. This has been sketched at the right-hands side of Fig. 1. The absence for the commensurate-incommensurate transition at the MNP state will be further confirmed by our specific-heat analysis, as no trace for a transition at $T_{R}$ was observed. The suppression of magnetic order transitions has already been argued as a consequence of finite size effects^58–60^. Although finite-size effects are indeed affecting the magnetic state of the alloys (note the broadening of the magnetic peaks located between $18^{\circ }< 2\theta <25^{\circ }$, corresponding to the (000) and (110) reflections, as the MNP size reduces, revealing a progressive loss of the AF ordering), in this case, it is the increasing inhomogenous microstrain which is playing a key role in the lack of reorientation transition. Incommensurate structures are very subtle, meaning that, although slight deviations from commensurate phases are very common, transitions to a global inconmmensurate magnetic structure are less favored^61^. Therefore, it should not be surprising that the existence of inhomogeneous microstrain within the nanoparticles, including the nanoparticle core, may provoke slight deviations from a pure inconmmensurate arrangement. What is more, these deviations from a pure incommensurate phase, even if minor, already exist at bulk state (5% kept the commensurate structure), and are enhanced in the nanoparticle state, preventing the magnetic moments to transition from a pure commensurate to a inconmmensurate magnetic arrangement.

The middle panels (Fig. 1b,d,f) depict the evolution of the magnetic moment *M* with temperature. These data were obtained as $M = \frac{\pi}{4}\mu _{\tau}$ and $M = \frac{3\pi}{4} \mu _{3\tau }$ for the fundamental (left axis) and third-harmonic contribution (right axis), respectively. It should be noted that, when the magnetic structure changes from the commensurate to the incommensurate arrangement at $T_R = 4.5$ K (and only for the bulk alloy), the $3\tau$ contribution vanishes. Following the loss of AF order revealed by the broaden magnetic peaks (see Fig. 1c,e), a drop in the value of the magnetic moment with decreasing MNP size can be detected. This is well-understood as the amount of AF-coupled entities decreases progressively along with the size reduction. The comparison with the Brillouin function calculated with a total angular momentum of $J=9/2$ (red dotted line) shows a reasonable agreement, as it has been already reported in other $\hbox {RCu}_{2}$ MNP systems^22,47^.

In order to access the low-*q* region (to better define the magnetic structure of $\hbox {NdCu}_{2}$ bulk and MNPs), we have performed small-angle neutron scattering (SANS) measurements. Figure 1g displays the experimental total (nuclear and magnetic) differential SANS cross section $d\Sigma / d\Omega$ as a function of the momentum transfer *q*. There, it can be observed the onset of a structure, which diffracts as a peak, at $T = 2$ K and $q \cong 2.93\,\hbox {nm}^{-1}$, only for the bulk alloy. This *q*-value corresponds to a real-space distance of about 2.14 nm, which is nearly 5 times the *a* parameter of the crystallographic unit cell^39,43^. This finding reveals that the RKKY-interaction-mediated spin structures are correlated up to a length scale corresponding to 5 nuclear cells. Complementary $\chi _{AC}(T)$ measurements on the bulk alloy (see Supplementary Fig. S6 in Ref.^57^) further support the existence of such a correlation, as the presence of a cusp in the $\chi ''(T,f)$ component at $T \lesssim T_{N}$ reveals that a large number of individual magnetic moments exhibit a different spin–spin correlation than that of the long-range AF-ordered spin. These short-range AF interactions are robust, since this peak remains almost unaffected even when a field of 3 T is applied (see supplementary figure Fig. S3 in Ref.^57^). The fact that this contribution is wiped out for the MNPs indicates that the size reduction towards the nanoscale destroys this additional correlation.

Unfortunately, in the present SANS experiments, we could only access large momentum-transfer values *q*, meaning that we could not measure the structure factor over the whole range of scattering vectors, necessary to also establish the AFM/SG structure via SANS, similar to what has been done on iron oxide, cobalt ferrite, and manganese–zinc–ferrite nanoparticles in Refs.^18,21,62^. Therefore, the present analyses will be constrained to the high-*q* peak, that was only present for the bulk alloy. The magnetic nature beneath the SANS peak observed for the bulk alloy is further confirmed by monitoring the change in $d\Sigma / d\Omega$ as the different magnetic states are traversed. This way, by changing the temperature from the commensurate structure ($T = 2.1$ K) to the incommensurate phase ($T = 4.8$ K), and then, to the paramagnetic state ($T > 6.5$ K), it can be observed how the peak found at $q \cong 2.93$ nm$^{-1}$ moves towards lower momentum transfers ($q \cong 2.61$ nm$^{-1}$) and softens (see left inset in Fig. 1g). This indicates that the transformation from a commensurate to an incommensurate phase also implies a change of the short (additional) correlation length, which now correlates spin structures up to a length scale corresponding to 6 nuclear cells. This is in good agreement with the enlarged magnetic cell of the incommensurate phase, which grows from 10 to 23 crystallographic unit cells, driven by the change of the propagation vector from $\tau = (0.6, 0, 0)$ to $\tau = (0.612, 0.042, 0)$^43^. Both SANS contributions corresponding to the magnetic state ($T = 2.1$ K and $T = 4.8$ K) are well below the one corresponding to the paramagnetic phase ($T = 11.3$ K). This is expected, since a rise in the scattering signal due to the increasing disorder should be recovered in the nonmagnetic state^63^.

Summarizing this section, the diffraction results unambiguously reveal that the core of the nanoparticle arranges following a commensurate structure, with no reorientation. In which concerns the surface ones, a magnetically disordered phase is taking place. The existence of interparticle correlations observed in the neutron diffraction patterns, together with the evidenced SG state in $\hbox {NdCu}_{2}$ MNPs^39^ allow us to state such a magnetically-disordered SG-like phase for these outer magnetic moments.

### Rejuvenation and memory effect measurements

**Figure 2:**
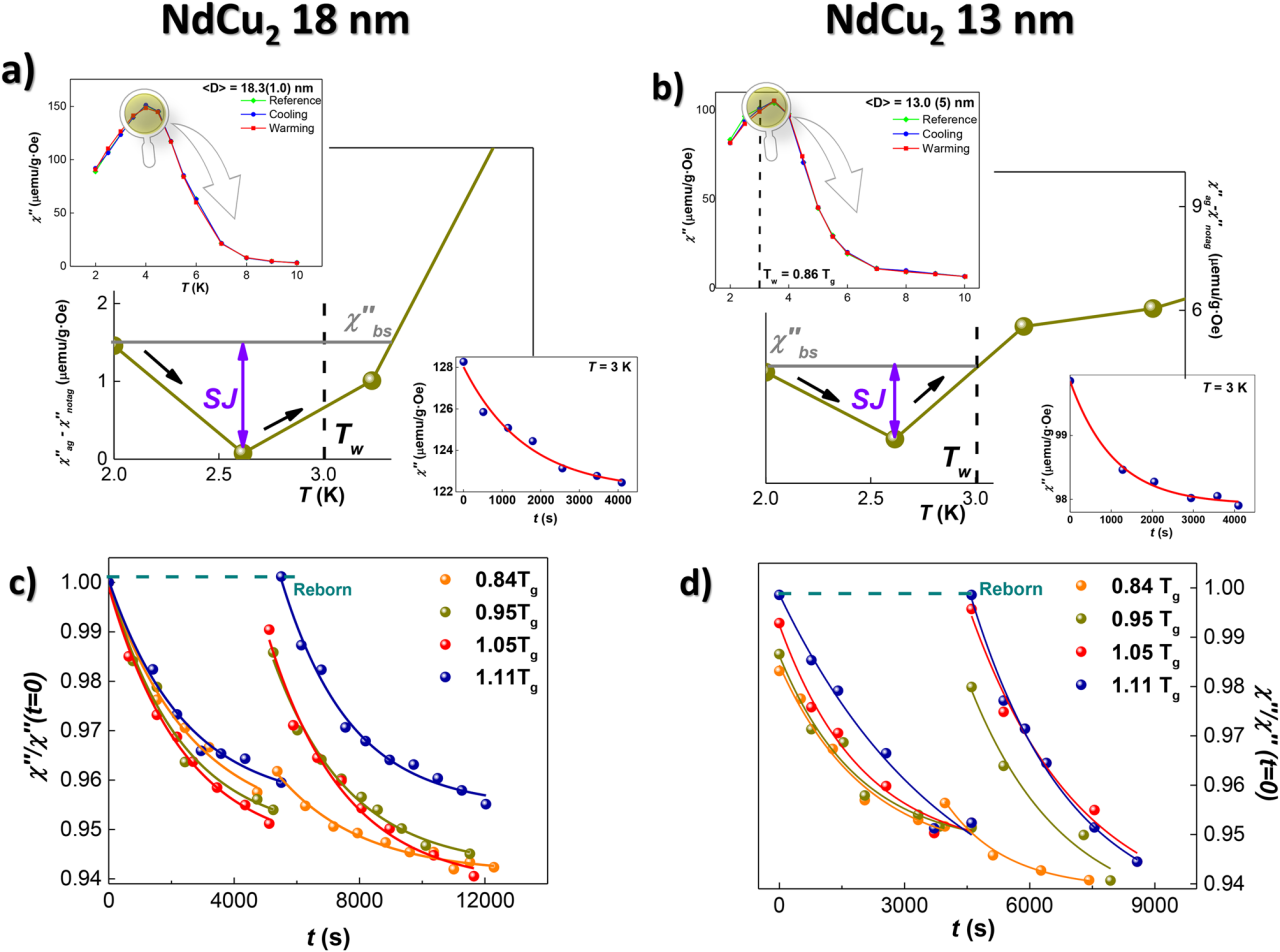
AC susceptibility results. Memory effects and aging phenomena measured at $f= 0.2$ Hz and $\mu _0 h_{ac} = 0.313$ mT for 18 nm-sized (**a**, **c**) and 13 nm-sized (**b**, **d**) $\hbox {NdCu}_{2}$ MNPs. In (**a**, **b**), the drop in $\chi ''_{ag} -\chi ''_{notag}$ at $T \lesssim$ $T_w$ (marked *SJ* in purple) evidences memory effects. Bottom insets in both figures depict the relaxation behavior of $\chi ''(t)$ at $T_w$. Note that the Y-axis are expressed in $\mu$emu/gOe. (**c**, **d**) depict $\chi ''(t)$ measured at $T_w$ before and after applying $4 \Delta T$ (cycles). Note that the SG state is completely reborn for $\Delta T = 1.11 \, T_g$.

In order to quantify the robustness of the SG phase settled for the magnetic moments located at the surface, we have analyzed the time-dependent $\chi _{AC}(t)$ response. Given that these phenomena are better determined on the out-of-phase component, we will restrict our analyses to the $\chi ''(T, t)$. We refer the reader to supplementary Fig. S7 for a brief comment on the in-phase component of the MNPs. Figure 2 includes the memory effect (Fig. 2a,b) and temperature-cycling protocols (Fig. 2c,d) corresponding to the 18 nm [(a) and (c)] and the 13 nm-sized [(b) and (d)] MNPs, respectively. The measurements are focused on the temperature region $T < 10$ K, given that both MNP ensembles enter the paramagnetic region already at $T = 7$ K^39,64^. The glass temperature $T_g$ of these MNPs, determined according to the emergence of a peak in the out-of-phase $\chi ''(T,f)$ component [see top insets in (a) and (b)], was located at $T_g = 3.8(1)$ K for both particle sizes. Therefore, the waiting temperature $T_w$ was fixed at $T_w = 3$ K, which corresponds to $T_w = 0.796 \approx 0.8\, T_g$. As can be deduced from Fig. 2a and b, memory effects appear when a stop is made at $T_w = 0.8 \, T_g$, as a drop is measured for the difference between the out-of-phase $\chi _{AC}$ component measured on cooling for the following two scenarios: (i) waiting at $T_w$ for about $10^{4}$ s and then resuming the cooling down to $T = 2$ K (denoted as $\chi ''_{ag}$), and (ii) cooling without waiting at $T_w$ (denoted as $\chi ''_{notag}$). The drop in the $\chi ''_{ag} - \chi ''_{notag}$ difference data is revealed for $T \lesssim T_g$ in both MNP sizes. The relaxation of $\chi ''(t)$ (bottom insets) follows the same trend as the one found in SGs^65–67^. There, a faster decay can be observed for the smallest MNPs (13 nm).

With the aim of accessing more information about the robustness of the magnetically frustrated and disordered SG surface moments, temperature cycles have been performed, following the protocol described in the “Magnetic characterization” subsection of the “Methods” section. Figure 2c and d include such measurements, performed at $T = 3$ K with cycles of $\Delta T =$ 0.84, 0.94, 1.05, and 1.11$\, T_g$. As it can be seen, the smaller the $\Delta T$, the slower the relaxation (decay). An interpretation of this effect is based on the results of the droplet model for SGs^68^. Within this framework, the correlated spins are assumed to form a *droplet* (or, equivalently, a *domain*) of a certain length, which behaves independently from the rest of the spins^69^. In our case, the slower relaxation implies that the *domains* (droplets) of correlated spins are larger^66^. Paying attention to the rise in the $\chi ''(t)$ value after having resumed the temperature rise (*i.e.*, $t > t_{\Delta T}$), the effect of a finite domain length scale $\ell _{\Delta T}$ can already be seen at $\Delta T = 0.84 \, T_g$, as the $\chi ''(t=t_{\Delta T})$ increases with respect to $\chi ''(t \lesssim t_{\Delta T})$. This rise is enhanced as the step $\Delta T$, until the initial value is met, i.e., $\chi ''(t=t_{\Delta T}) = \chi ''(t=0)$. The situation can be explained taking into account that the magnetic moments freeze at $t = 0$ in some particular disordered configuration (domain). Such a configuration is imposed by the frustration of the oscillatory RKKY interactions. Thus, a particular *metastable* initial spin configuration of the droplet is established. When $\Delta T$ is increased, the larger droplets will start to break down, while a new overall droplet configuration appears. In this way, at least two SG domain configurations coexist, implying the existence of different length scales (conversely, time scales). It is noticeable that in our case, the spin domains are located at the nanoparticle surface, arranged quasi-spherically as a whole, while previous evidence have been reported in well-defined SGs (e.g., the Ising system $\hbox {Fe}_{0.5}\hbox {Mn}_{0.5}\hbox {TiO}_{3}$^70^ or the Heisenberg-like material $\hbox {CdCr}_{1.7}\hbox {In}_{0.3}\hbox {S}_{4}$^66^). Spin-glass correlations in the $\hbox {NdCu}_{2}$ MNPs exist even at temperatures above $T_g$, as $\chi ''(t=t_{\Delta T})$ is still below $\chi ''(t=0)$ for $\Delta T = 1.05 \, T_g$. This underlines the robustness of the SG interactions. Then, when $\Delta T = 1.11 \, T_g$, the energy barriers of the larger initial clusters become smaller compared to the thermal energy, meaning that the domain landscape established at $T = 3$ K prior to the temperature cycling is completely destroyed, and thus, the SG state is said to be completely reborn (*rejuvenation*), as it has already been showed in other ensembles of MNPs evidencing complex magnetic structures (e.g., $\hbox {SrFe}_{12}\hbox {O}_{19}$^54^). This rejuvenation of the moments sitting at the surface of the MNPs is somehow surprising, considering that the coupling between neighboring moments is not so well defined, compared to the archetypal Ising or Heisenberg systems mentioned above, where the aging and rejuventation analyses have been carried out in great detail^71–73^.

It is worth mentioning that both memory and temperature cycling measurements have been performed for the bulk alloy as well in order to check whether the incommensurate-to-commensurate transition of the magnetic structure could imply frustration that might give rise to a SG state. Not surprisingly, neither traces of memory effects nor relaxation effects are evidenced in the bulk alloy at any temperature surrounding $T_R = 4.5$ K. This ensures that the transition from the commensurate phase to the incommensurate one is accomplished without the weakening of any magnetic AF order, as the magnetic moments keep well-aligned in the square-up structure.

In order to put into context the onset of memory effects connected to SAF MNPs, we have worked out a simple procedure to compare the robustness of such effects among different SG systems. For this purpose, we define the SG rejuvenation jump (*SJ*) parameter as follows:

1 $$SJ = \left|\frac{\chi''_{memory}-\chi''_{bs}}{\chi''_{bs}}\right|,$$

where $\chi ''_{memory} = \chi ''_{ag} - \chi ''_{notag}$, and $\chi ''_{bs}$ is defined as the $\chi ''_{memory}$ value corresponding to the temperature at which memory effects emerge (see gray line in Fig. 2a,b) . We have estimated also the *SJ* values corresponding to the canonical SGs CuMn ($SJ=7.5$) and $\hbox {CdCr}_{1.7}\hbox {In}_{0.3}\hbox {S}_{4}$ ($SJ=12$)^66,74^, and also for SG NiFe nanoparticles of 12 nm size embedded in a SiO$_{2}$ matrix ($SJ=0.8$)^52^. Our $\hbox {NdCu}_{2}$ ensembles showcase $SJ = 0.5$ and $SJ = 0.6$ for 18 nm and 13 nm-sized MNPs, respectively. As it can be seen, these values are smaller than those of the canonical bulk SGs. This can be readily understood as the magnetically-frustrated RKKY interactions are better accomplished in the bulk systems. where the unit cell distortion and microstrain are minim. But compared to other nanoparticle ensembles, the values obtained for our $\hbox {NdCu}_{2}$ MNPs are in good agreement with this of NiFe nanoparticles of a similar size. Here, we can comment on the positive tendency for the *SJ* values as the nanoparticle size decreases. This effect can be understood in terms of an increasing surface-to-core ratio, i.e., the amount of magnetically-frustrated moments increases when reducing the nanoparticle size, which easily translates into the enhancement of memory effects.

### Specific heat measurements

Specific heat measurements have been performed in order to complete the information about the magnetic transitions undergone by the $\hbox {NdCu}_{2}$ ensembles. Following common practice (e.g., Refs.^56,75,76^), $c_P$ is assumed to consist of three additive contributions:

2 $$\begin{aligned} c_P = c_{ph} + c_{el} + c_{mag} , \end{aligned}$$

where $c_{ph}$ is the phonon contribution, which is assumed to obey a Debye model, $c_{el}$ is the electronic term (depending linearly on *T*), and $c_{mag}$ represents the magnetic contribution, including the effect of the crystalline electric field (CEF). To extract the $c_{mag}$ term, we have combined both $c_{el}$ and $c_{ph}$ contributions into a single term, labeled as $c_{lattice}$, which has been estimated taking into account the existence of two different symmetry environments: one for the magnetic moments located within the core, and one for those located at the nanoparticle surface. To separate both contributions, we have employed the procedure explained in Ref.^39^, where $c_{lattice}$ is taken from the nonmagnetic isostructural $\hbox {YCu}_{2}$ compound^77,78^, including a renormalization factor to account for the different molar masses between the magnetic ($\hbox {Nd}^{3{+}}$) and nonmagnetic ($\hbox {Y}^{3{+}}$) ions^75^. The surface contribution to $c_{lattice}$ is fitted according to the phonon and electronic contributions. Indeed, the latter ones are weighted by a factor $N_c$ that accounts for the core-to-volume ratio^39^. In this way, the $c_{lattice}$ contribution is calculated as

3 $$\begin{aligned} c_{lattice} = N_{c} \left[ \gamma _{c} T + 9R \left( \frac{T}{\theta _{D}^{c}} \right) \int _{0}^{\theta _{D}^{c}/T} dx \frac{x^{4} e^{x}}{( e^{x}-1)^{2}} \right] + N_{s} \left[ \gamma _{s} T + 9R \left( \frac{T}{\theta _{D}^{s}} \right) \int _{0}^{\theta _{D}^{s}/T} dx \frac{x^{4} e^{x}}{( e^{x}-1)^{2}} \right] , \end{aligned}$$

where $R = 8.314$ J/(K mol), and $\theta _{D}^{c}$ and $\theta _{D}^{s}$ denote the Debye temperatures corresponding, respectively, to the core and surface contributions. The fraction of magnetic moments located within the core, $N_c$, is 0.47 and 0.34 for the 18 nm and 13 nm-sized MNPs, respectively (indeed, the surface moments, $N_s = 1-N_c$). We refer the reader to Supplementary Section “Estimating the core-to-volume ratio” for a brief comment on the estimation of the $N_c$ values.

**Figure 3:**
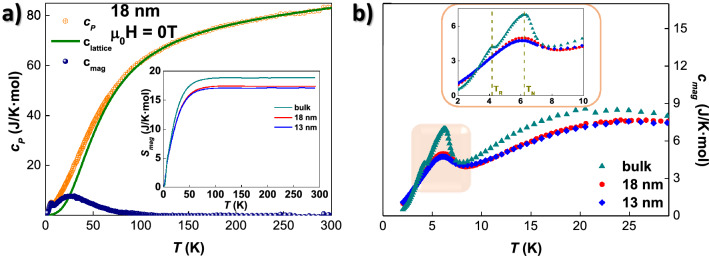
Zero-field specific-heat results. (**a**) Experimental $c_P$ (orange), calculated $c_{lattice}$ (green) and $c_{mag}$ (blue) contributions corresponding to 18 nm-sized $\hbox {NdCu}_{2}$ MNPs. Inset compares $S_{mag}$ calculated for bulk (dark cyan), 18 nm (red), and 13 nm-sized (blue) MNPs. (**b**) $c_{mag}$ contribution of bulk, 18 nm, and 13 nm-sized MNP alloys. The inset provides a closer view on the magnetic transition region, where the positions of the Néel ($T_N$) and reorientation ($T_R$) temperatures have been marked by dashed vertical lines.

Following the above-mentioned fitting procedure, Fig. 3a showcases the measured $c_P$ and the calculated $c_{lattice}$ and $c_{mag}$ contributions for the 18 nm-sized MNP ensemble. There, it can be seen how the calculated $c_{lattice}(T =300\,\hbox {K}) =83.01\,\hbox {J}/(\text {K mol})$ matches well the expected value according to the Dulong–Petit law^79^. Values for $\gamma ^{bulk} = 12.14(13)$ mJ/($\text {mol K}^{2}$) and $\theta _{D}^{bulk} = 224.7(6)$ K have been obtained, in good agreement with the ones reported for polycrystalline bulk $\hbox {NdCu}_{2}$^56^. In the case of the MNP surface, $\gamma ^{s} = 22.82(2)$ mJ/($\text {mol K}^{2}$) and $\theta _{D}^{s} = 281(4)$ K for 18 nm, and $\gamma ^{s} =21.5(5)$ mJ/($\text {mol K}^{2}$) and $\theta _{D}^{s} = 240(6)$ K for 13 nm MNPs, values that are greater compared to the bulk ones, as expected^22,37,39^. The inset in Fig. 3a includes the magnetic entropy $S_{mag}$, which has has been calculated as follows:

4 $$\begin{aligned} S_{mag}^{exp} = \int _{0}^{300} \frac{c_{mag}}{T} dT \end{aligned}$$

It can be seen, on the one hand, that the magnetic entropy of the bulk alloy, $S_{mag}^{exp} = 18.8(1)$ J/(K mol), lies slightly below the theoretical $S_{mag}^{theo}(300 \, \mathrm {K}) = R [\log (2J+1)] = 19.14\,\hbox {J}/\hbox {Kmol}$^79^, so does compared to the MNPs, where $S_{mag}^{exp} \approx$ 17 J/Kmol. This reduction might be caused by the existence of short-range correlations and of a distorted surface, which both have been claimed to keep the $S_{mag}^{exp} <S_{mag}^{theo}$^22,39,56,64^. Additionally, the value of $S_{mag}^{exp}$ around $T_N$ for the MNPs is about 3.6 J/Kmol, which is also below the expected value $S_{mag}^{theo}(T_N) = R [\log (2)] =$ 5.76 J/(Kmol) for a complete removal of the two-fold spin degeneracy of the CEF ground-state doublet^80^. The same picture holds for the bulk alloy, where $S_{mag}^{exp} \cong$ 4.39 J/(K mol). These facts (reduced $S_{mag}^{exp}$ at 300 K and $T_N$) were also found in polycrystalline bulk $\hbox {NdCu}_{2}$ and $\hbox {CeCu}_{2}$ alloys^56,64^.

Figure 3b shows the $c_{mag}$ contribution corresponding to the three alloys at zero external field, while the $c_{mag}$ obtained upon applying different magnetic fields can be inspected in Supplementary Fig. S8. The region nearby the low-temperature magnetic transitions has been enlarged in the inset to observe in closer detail the lack of a reorientation transition in the MNPs. Thereby, the $c_{mag}$ reveals clearly how the bulk AF state survives in the MNPs, according to the occurrence of an AF $\lambda$-anomaly in the MNPs. The $T_{N}$ value is not affected by the nanoscaling, as it is kept constant at $T_N = 6.2(1)$ K. This is in good agreement with what has already been reported in Ref.^39^. The most interesting change to be noticed with respect to the bulk alloy is the lack of a reorientation transition, which confirms the ND results, where the magnetic structure of the AF core moments follows a commensurate square-up antiferromagnetic structure in the whole magnetic region. The Schottky-like contribution found above $T_N$ is not related to extra magnetic transitions, yet it arises from CEF effects. This reveals, in the same way as in Ref.^39^, that the energy level schemes are maintained in the nanoparticle regime in a way very similar to the bulk. A complete explanation for this finding can be found in Ref.^39^, where the joint analyses between specific heat and inelastic neutron scattering unveiled the prevalence of the CEF schemes despite the size reduction to the nanoscale.

## Conclusion

Two MNP ensembles of $\hbox {NdCu}_{2}$ with 18 nm and 13 nm particle size have been produced and characterized in order to scrutinize the dual spin dynamics driven by the core and surface environments. The core magnetic moments arrange into a commensurate square-up antiferromagnetic structure up to $T_N$ without exhibiting any reorientation to an incommensurate phase, as it was the case for the bulk alloy. On the other hand, the magnetic moments located at the MNP surface give rise to a collective spin-glass phase, for which we have been able to determine quantitatively the time-dependent memory effects and aging phenomena. Such magnetically-frustrated correlations have been compared with those found in canonical and MNP spin glass phases by means of the *SJ* parameter. A strong reduction in the *SJ* value has been determined when passing from ideal Ising spin-glass systems to superantiferromagnetic MNPs. Finally, although $\hbox {NdCu}_{2}$ MNPs adopt an AF/SG core/surface arrangement very similar to the one reported in $\hbox {Cr}_{2}\hbox {O}_{3}$, NiO or CoO–Pt MNPs^40–42^, the magnetization dynamics of the presented $4f\ \hbox {NdCu}_{2}$ MNPs is radically different from the one of transition metal-based MNPs, as the magnetically-frustrated moments play a key role in the global MNP magnetic dynamics response. Therefore, our work shows how the complex magnetic structure of bulk $\hbox {NdCu}_{2}$ evolves with decreasing particle size, as well as it provides a depiction on how the size-effects modify the magnetically-frustrated RKKY exchanged interactions taking place at the nanoparticle surface.

## Methods

### Sample production

Polycrystalline $\hbox {NdCu}_{2}$ pellets have been obtained by melting the constituents in an arc furnace (MAM-1, Johanna Otto GmbH) under an Ar atmosphere (99.99%). The resulting alloy was sealed-off in a glove box under Ar pressure (99.99%) to avoid oxidation, and grinded for times of 2h and 5h in a Retsch PM 400/2 high-energy planetary ball mill (using WC containers). This technique allows to obtain easily a large amount ($\sim$ 5 g) of MNPs.

### Neutron diffraction and small-angle neutron scattering

The microscopic structure of the $\hbox {NdCu}_{2}$ samples was studied using neutron diffraction (ND) and small-angle neutron scattering (SANS). ND measurements have been carried out at the G4.1 instrument (LLB, France) using a wavelength of $\lambda = 2.426$ Å at temperatures between 1.5 K and 15 K. A measuring time of 8 h for each diffraction pattern has been chosen to assure a high signal-to-noise ratio. SANS measurements were performed at the ZOOM instrument (ISIS, UK). The following parameters were employed: sample-to-detector distance: 4 m, wavelength range: 1.75 Å $< \lambda<$ 16.5 Å, sample temperatures: 2–100 K, applied fields: 0–3 T.

### Magnetic characterization

The magnetic characterization was performed by means of dynamic $\chi _{AC}(t, f, T)$ measurements in a QD-MPMS (SQUID) magnetometer located at the University of Cantabria, Spain. The ensembles of MNPs were measured between *T* = 2–300 K under an oscillating magnetic field amplitude of $\mu _0 h_{AC} =$ 0.313 mT and a frequency of $f= 0.2\,\hbox {Hz}$. To probe memory effects and aging phenomena, several protocols can be found in the literature, including both static $M_{DC}$ and dynamic $\chi_{AC}$ susceptibility measurements^55,66,70,81,82^. In this work, we have recorded the out-of-phase $\chi ''$ component of the dynamic $\chi _{AC}$ susceptibility, as it allows to detect in more detail the subtleties concerning the spin dynamics^55,70^. Briefly, in order to trace the memory effects, we have compared the difference between the AC susceptibility measured upon warming (i) without making any stop *(reference)*, and (ii) after having made a stop at the waiting temperature $T_{w}$ (*warming*) for $t>10^3$ s. The occurrence of a drop in this difference at temperature values slightly below $T_g$ will account for the memory effect. Concerning the aging phenomena, these are probed by inspecting the $\chi ''(t)$ data. Moreover, the *robustness* of the SG-like frustrated interactions has been further investigated by applying a *temperature cycling* protocol. This consists of measuring the $\chi ''$ *versus* *t* dependency at a certain $T_w$ value within the SG phase (in our case, $T_w \sim 0.8 \, T_g$) for a sufficiently long period of time ($t \sim 10^3$ s). Once the waiting time is over, the temperature has been raised by a certain $\Delta T$ up to temperature values close to that of $T_g$ ($\Delta T$ varies between 0.85 $T_g$ and 1.1 $T_g$). Immediately thereafter, the temperature is lowered down to $T_w$, and the $\chi ''(t)$ signal is measured again for $t \sim$ 10$^{4}$–10$^{5}$ s. This cycling protocol mimics the one already reported in Ref.^70^, and allows to monitor adequately the robustness of the SG phase.

### Specific heat measurements

The thermodynamic properties have been studied by means of heat capacity measurements. These were performed using a QD-PPMS instrument (University of Cantabria) in the temperature range between 2 and 300 K, under zero applied magnetic field, and at magnetic fields of 1 T and 8 T. Measurements were performed following the relaxation method^83^.

## Supplementary Information

Supplementary Information.

## Data Availability

All data generated or analyzed during this study are included in this published article and its supplementary information files.
