# Supplementary material for: Magnetic order and disorder environments in superantiferromagnetic NdCu2 nanoparticles
Source: Sci Rep. 2022 Jun 13;12:9733. doi: 10.1038/s41598-022-13817-7 (PMC9192703; doi:10.1038/s41598-022-13817-7)
Supplement: Supplementary file 1 — Supplementary Information. [file 41598_2022_13817_MOESM1_ESM.pdf]

Supplementary Material to: Magnetic Order and Disorder Environments in Superantiferromagnetic $NdCu_2$ Nanoparticles

E. M. Jefremovas[1,*], P. Svedlindh[2], F. Damay[3], D. Alba Venero[4], A. Michels[5], J. A. Blanco[6], and L. Fernández Barquín[1]

[1]Department CITIMAC, Facultad de Ciencias, Universidad de Cantabria, 39005 Santander, Spain
[2]Department of Materials Science and Engineering, Uppsala University, Box 35, SE-751 03 Uppsala, Sweden
[3]Laboratoire Léon Brillouin, Université Paris-Saclay, CEA-CNRS, 91191 Gif–sur–Yvette Cedex, France
[4]ISIS Neutron and Muon Facility, Rutherford Appleton Laboratory, Didcot, OX11 0QX, United Kingdom
[5]Department of Physics and Materials Science, University of Luxembourg, L–1511 Luxembourg
[6]Department of Physics, University of Oviedo, 33007 Oviedo, Spain
[*]martinjel@unican.es

ABSTRACT

This Supplementary Material includes supporting information on the microscopic structure of the nanoparticles. In this way, two representative TEM images have been included to access more information on the MNP morphology, as well as supplementary neutron diffraction (ND) and small–angle neutron scattering (SANS) data. We are also including the estimation of the geometrical core–to–volume ratio for both MNP ensembles (18 and 13 nm–sized). Supplemental static and dynamic magnetization measurements are displayed, accounting for the exchange bias effect, the dynamic in–phase and out–of–phase susceptibility of bulk $NdCu_2$, plus an overview of the in–phase $\chi'(T)$ component for the 18 and 13 nm–sized MNPs. Finally, the field dependence of the c_{mag} contribution to the specific heat is displayed.

Transmission Electron Microscopy analysis

Figs. S 1 (a) and (b) include two representative Transmission Electron Microscopy (TEM) images corresponding to $NdCu_2$ 5h–milled MNPs. The morphology of the MNPs can be considered as quasi–spherical, in good agreement with the previous morphology reported for $TbCu_2$ and $Tb_{0.5}Gd_{0.5}Cu_2$ MNPs produced by our group following the same route (ball milling)[1,2]. The statistical analyses of these images (19 nanoparticles counted) point to a mean nanoparticle size $\langle D \rangle = 12.6$ nm with size dispersion σ = 2.6 nm. This value is compatible with the ones obtained by means of both XRD [13.0(5)] and ND [12.7(1.0)] characterization, even if the number of MNPs counted is low.

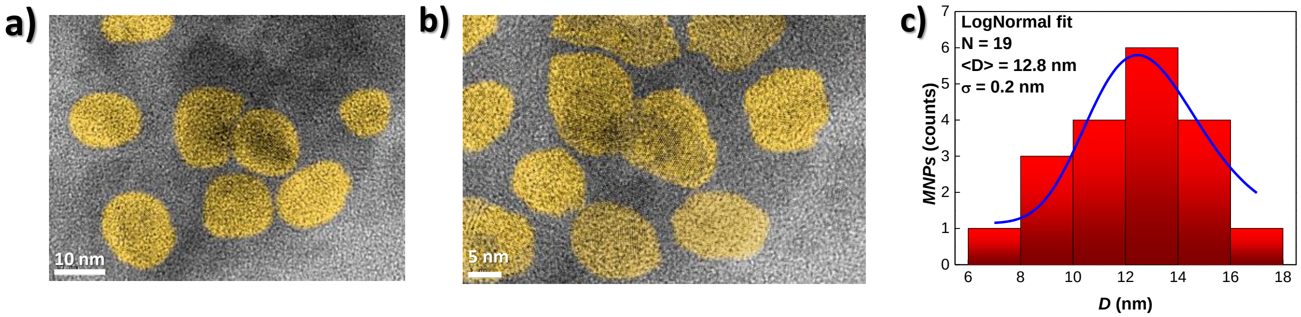

Fig. S 1. (a) and (b) representative TEM images corresponding to $NdCu_2$ 5h–milled MNPs, and (c) their corresponding size distribution ($N = 19$ MNPs). The fitting follows a LogNormal distribution, pointing to $\langle D \rangle = 12.6(3)$ nm.

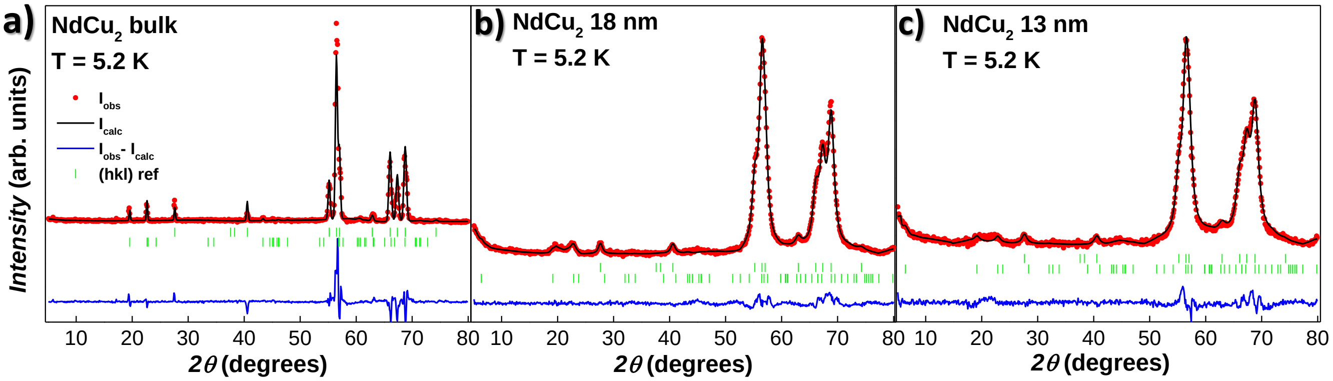

Fig. S 2. Neutron diffraction patterns for bulk $NdCu_2$ (a), 18 nm (b), and 13 nm-sized (c) MNPs measured between $5^\circ < 2\theta < 80^\circ$ at $T = 5.2$ K, *i.e.*, above the bulk reorientation temperature of $T_R = 4.5$ K. Red dots represent the experimental data, black lines are the calculated Rietveld refinements, and the blue lines at the bottom correspond to the difference between the calculated and experimental values. Green bars mark the position of the (hkl) positions of the nuclear (top) and magnetic (bottom) structures. The bulk magnetic structure is well–fitted according to an incommensurate description, while the MNPs (18 nm and 13 nm) maintain the commensurate square-up modulation at $T = 5.2$ K.

Neutron diffraction

Figure S2 represents the ND patterns corresponding to bulk (a), 18 nm (b), and 13 nm-sized (c) MNPs of $NdCu_2$. According to Ref. 3, the bulk magnetic structure at $T = 5.2$ K has already undergone a phase transition from a commensurate to an incommensurate structure with a propagation vector of $\tau = (0.612, 0.042, 0)$. All the Bragg peaks are well–fitted according to this description. On the other hand, the ND data in Fig. S2(b) and (c) maintain the commensurate structure. The rise in intensity in the low-angle region ($2\theta < 7^\circ$, $q < 0.316$ Å^{-1}) indicates the occurrence of interparticle correlations which can be connected to a surface spin–glass phase.

Small–angle neutron scattering

Figure S3 displays the field evolution of the total (nuclear and magnetic) SANS cross section $d\Sigma/d\Omega$ of bulk $NdCu_2$ in the neighborhood of the diffraction peak at $q \cong 2.93$ nm^{-1} (with $2\pi/2.93\,\text{nm}^{-1} \cong 2.14$ nm). These short–range AF interactions are rather robust, since the peak remains almost unaffected even when a field of 3 T is applied (see inset in Fig. S3).

Estimating the core–to–volume ratio

Following eq. S1 included in Ref.[4], the geometrical core–to–volume ratio corresponding to a MNP size of $\langle D \rangle$ =18 and 13 nm has been estimated as:

$$N_c = \frac{V_{core}}{V_{MNP}} = \left(\frac{D_{core}}{D_{MNP}}\right)^3 = \begin{cases} \left(\frac{14}{18}\right)^3 \approx 0.47 \\ \left(\frac{9}{13}\right)^3 \approx 0.33 \end{cases} \quad (1)$$

where the MNPs have been assumed to be spherical and a shell thickness of 2 nm is considered, as it is commonly found in MNPs[1] (see sketch in Fig. S4).

Exchange Bias effect

Fig. S 5 showcases the central region of the hysteresis loops performed on $NdCu_2$ 13 nm–sized MNPs. The sample has been cooled to $T = 3$ K with applying an external field of 5T. A slight negative shift of cycles can be detected, accounting for an exchange bias field $\mu_0 H_{EB} \sim 1.5$ mT. This value is two orders of magnitude below the one reported for transition metal–based MNPs, *e.g.*, $\mu_0 H_{EB} \sim 120$ and 300 mT for $CoFe_2O_4$ ($T_f \sim 125$ K)[5] or FeO/Fe_3O_4 ($T_N \sim 200$ K) MNPs[6]; and even below that of 3 mT reported for 8 nm–sized $TbCu_2$ MNPs[7]. The fact that $NdCu_2$ displays extremely low values for its ordering ($T_N \sim 6.5$ K) and freezing ($T_f \sim 5.5$ K) transitions hampers the observation of large exchange bias field.

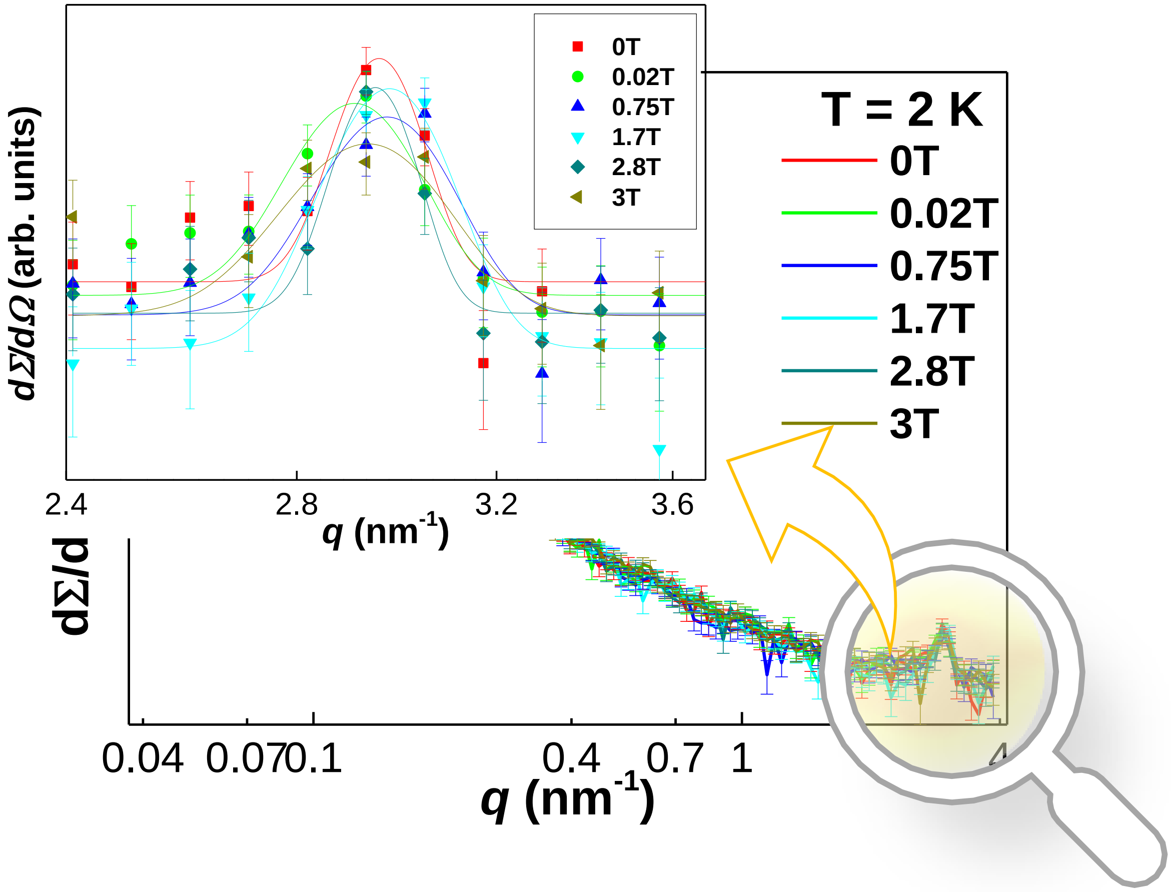

Fig. S 3. Field–dependence ($\mu_0 H$ between 0 and 3 T) of $d\Sigma/d\Omega$ of bulk $NdCu_2$ at $T = 2$ K (log–log scale). The inset enlarges the q–region in the vicinity of the magnetic diffraction peak at $q \cong 2.93$ nm^{-1}.

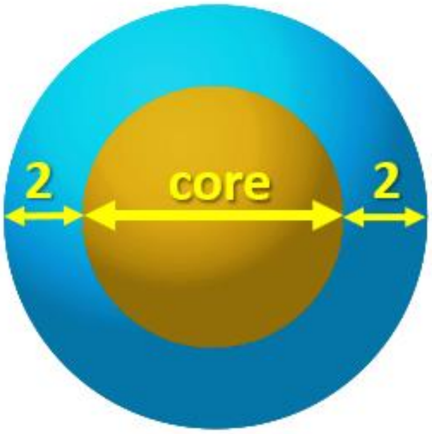

Fig. S 4. Schematic representation of the nanoparticles, where the shell is considered to be of 2 nm thickness.

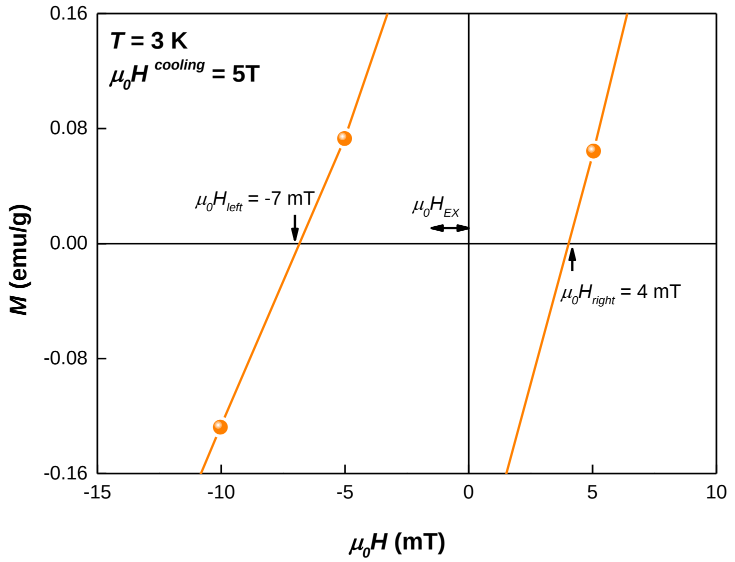

Fig. S 5. Central region of the hysteresis loops performed on 13 nm–sized $NdCu_2$ MNPs. An exchange bias field of $\mu_0 H_{EB} \sim$ 1.5 mT can be observed.

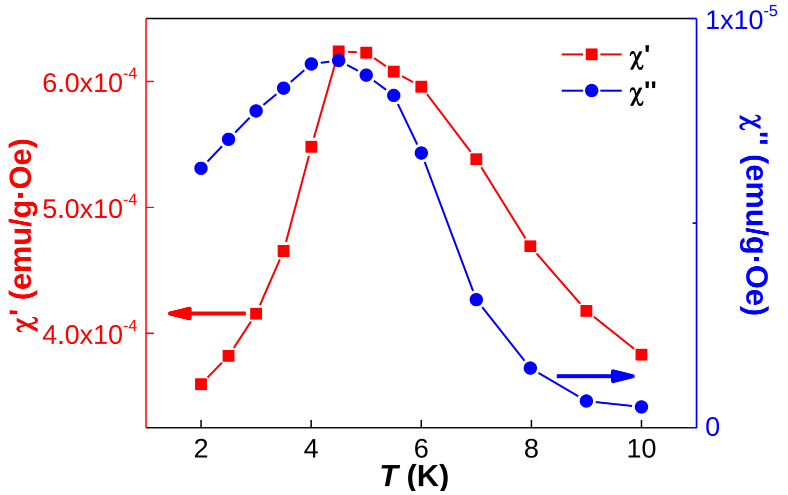

Fig. S 6. In–phase χ' (red) and out–of–phase χ'' (blue) components of the dynamic susceptibility $\chi_{AC}(T)$ measured at $f = 0.2$ Hz and $\mu_0 h = 0.313$ mT for $NdCu_2$ polycrystalline bulk alloy.

Dynamic susceptibility of bulk $NdCu_2$

Fig. S 6 the in–phase and out–of–phase components for the bulk $NdCu_2$ alloy. The in–phase shows two peaks, at $T \sim 4.5$ and $T \sim 6.5$ K, which correspond to the T_R and T_N transitions. The out–of–phase displays a broad peak centered at $T \sim 4$ K.

In–phase $\chi'(T, f)$ component of $NdCu_2$ MNPs

Fig. S 7 includes the dynamic susceptibility in–phase $\chi'(T, f)$ component corresponding to the 18 and 13 nm–sized MNPs, respectively. There, it can be seen the occurrence of a broad peak in the vicinity of $T \approx 5$ K for both MNP ensembles. This maximum have evidenced a shift towards higher temperatures when increasing the frequency[4], which is a characteristic behavior of freezing transitions[8,9]. Although additionally to this SG–cusp, a maximum in $\chi'(T, f)$ corresponding to the AF Néel transition should be observed at $T = T_N$, only a single broad peak is detected, as a consequence of the proximity of both T_N and T_f transitions.

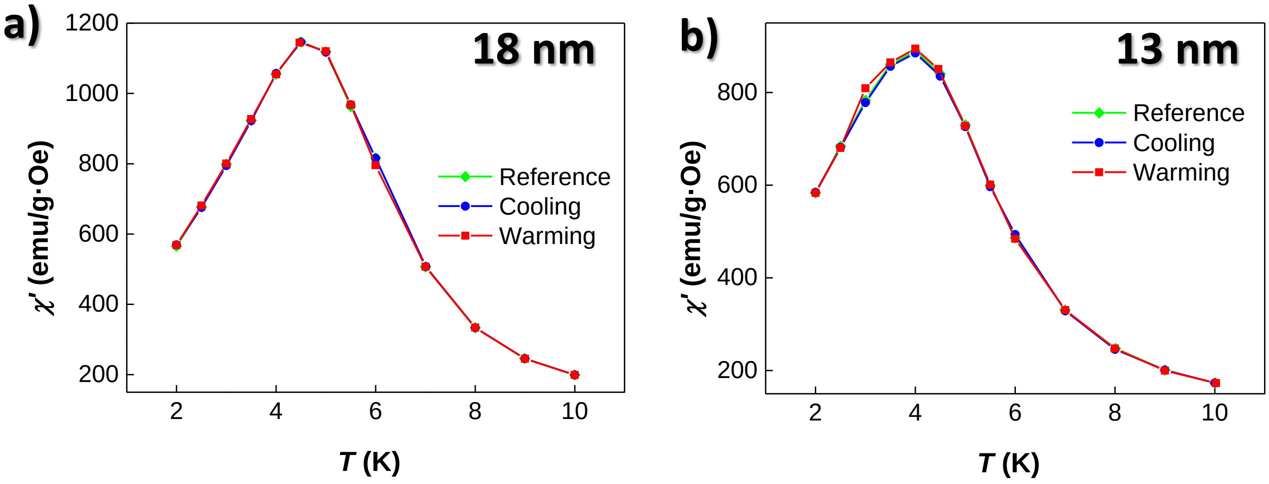

Fig. S 7. In–phase $\chi'(T)$ component corresponding to (a) 18 and (b) 13 nm–sized $NdCu_2$ MNPs measured under f =0.2 Hz and h =0.313 mT.

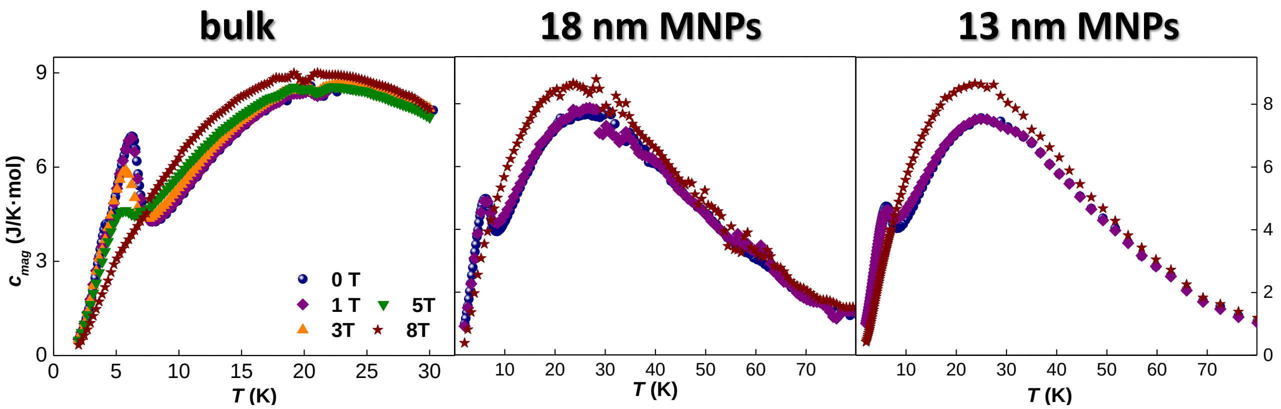

Fig. S 8. Magnetic contribution to the specific heat, c_{mag}, as a function of temperature T for bulk (a), 18 nm (b), and 13 nm-sized (c) MNPs measured under external magnetic fields of 0, 1, and 8 T. Additionally, for the case of the $NdCu_2$ bulk alloy, data at 3 T and 5 T are shown.

Magnetic contribution to the specific heat

Figure S8 shows the field dependence of the magnetic contribution to the specific heat, c_{mag}, corresponding to bulk (a), 18 nm-sized (b), and 13 nm-sized MNPs (c). First of all, it should be mentioned that, as for the case of the 13 nm-sized MNPs[4], for which $\gamma^s = 21.5(5)$ mJ/(mol K^2) and $\theta_D^s = 240(6)$ K were obtained[4], the 18 nm-sized MNPs also do evidence alterations in the surface propagation, in the form of a softening of $\gamma^s = 22.82(2)$ mJ/(mol K^2) and $\theta_D^s = 281(4)$ K. These values are, as expected, larger than the bulk ones, which have been obtained as $\gamma^{bulk} = 12.14(13)$ mJ/(mol K^2) and $\theta_D^{bulk} = 224.7(6)$ K, in good agreement with the ones reported in Ref. 10.

The field dependence of the c_{mag} contribution allow to detect how the Néel transition is almost destroyed at an applied field of $\mu_0 H = 3$ T for the bulk alloy [Fig. S8(a)], which is congruent with the existence of a metamagnetic transition found at 2.7 T[11]. According to both the modeling in Ref. 11 and the experimental magnetization study presented in Ref. 3, the magnetic moments are, above 2.7 T, ferrimagnetically–oriented. The fact that we could still recover a slight hump, even at 8 T, underlines the robustness of the AF–coupling, that still exists for some magnetic entities within the global F1 phase. In the case of the MNPs [Fig. S8(b) and (c)], the situation is very similar: the Néel transition is barely affected at 1 T, whereas it is completely wiped out at 8 T, in good agreement with $M(H)$ results[4].

References

1. Echevarria-Bonet, C. *et al.* Size-induced superantiferromagnetism with reentrant spin-glass behavior in metallic nanoparticles of $TbCu_2$. *Phys. Rev. B* **87**, 180407(R) (2013).
2. Jefremovas, E. M. *et al.* Exploring the Different Degrees of Magnetic Disorder in $Tb_xR_{1\text{-}x}Cu_2$ Nanoparticle Alloys. *Nanomaterials* **10**, 2148 (2020).
3. Arons, R., Loewenhaupt, M., Reif, T. & Gratz, E. The magnetic structures of $NdCu_2$ in zero field. *J. Phys.: Condens. Matter* **6**, 6789 (1994).
4. Jefremovas, E. M. *et al.* Observation of surface magnons and crystalline electric field shifts in superantiferromagnetic $NdCu_2$ nanoparticles. *Phys. Rev. B* **104**, 134404 (2021).
5. Mumtaz, A., Maaz, K., Janjua, B., Hasanain, S. & Bertino, M. F. Exchange bias and vertical shift in $CoFe_2O_4$ nanoparticles. *J. magnetism magnetic materials* **313**, 266–272 (2007).
6. Sun, X., Frey Huls, N., Sigdel, A. & Sun, S. Tuning exchange bias in core/shell FeO/Fe_3O_4 nanoparticles. *Nano letters* **12**, 246–251 (2012).
7. Echevarria-Bonet, C. *et al.* Magnetic phase diagram of superantiferromagnetic $TbCu_2$ nanoparticles. *J. Phys.: Condens. Matter* **27**, 496002 (2015).
8. Mydosh, J. A. *Spin glasses: an experimental introduction* (CRC Press, 2014).
9. Zhou, G. & Bakker, H. Spin-glass behavior of mechanically milled crystalline $gdal_2$. *Phys. Rev. Lett.* **73**, 344 (1994).
10. Gratz, E. *et al.* Structural, magnetic, electronic and transport properties of $NdCu_2$. *J. Phys.: Condens. Matter* **3**, 9297 (1991).
11. Ellerby, M., McEwen, K., De Podesta, M., Rotter, M. & Gratz, E. Detailed magnetization study of $NdCu_2$ at low temperatures. *J. Phys.: Condens. Matter* **7**, 1897 (1995).
